# A dataset of attributes from papers of a machine learning conference

**DOI:** 10.1016/j.dib.2019.103836

**Published:** 2019-03-26

**Authors:** Diego Vallejo-Huanga, Paulina Morillo, Cèsar Ferri

**Affiliations:** a Universidad Politécnica Salesiana, Research Group IDEIAGEOCA, Quito, Ecuador; b Universidad San Francisco de Quito, Department of Mathematics, Quito, Ecuador; c Universidad de las Américas, Department of Physics and Mathematics, Quito, Ecuador; d Universitat Politècnica de València, DSIC, València, Spain

**Keywords:** Scientific documents, Machine learning

## Abstract

In this work, we present a dataset which provides information on the scientific program of a set conferences of Machine Learning. Data were extracted from the IEEE Xplore Digital Library and the official web site of the International Conference on Machine Learning Applications (ICMLA). We include data of four different editions (from 2014 to 2017). Web scrapping techniques were used to mine the data contained in these web sites. The dataset covers 448 papers presented in the conference and every paper contains 6 attributes including information about the thematic session in which they were presented in the conference. The dataset is hosted in the Mendeley Dataset Repository.

**Table:** Specifications table

Subject area	*Computer Science*
More specific subject area	Artificial Intelligence, Machine Learning, Text Mining, Natural Language Processing, Clustering Analysis, Classification Analysis
Type of data	Table in CSV format
How data was acquired	Web Scrapping from different sources
Data format	Raw, analyzed
Experimental factors	The articles were classified according to the thematic sessions articulated by the organizers of the conference
Experimental features	The dataset contains information about the accepted papers of the International Conference on Machine Learning Applications (ICMLA), editions 2014, 2015, 2016 and 2017, extracted with web scrapping techniques from the IEEE Xplore Digital Library.
Data source location	United States
Data accessibility	Vallejo-Huanga, Diego; Morillo, Paulina; Ferri, Cèsar (2018), “ICMLA 2014/2015/2016/2017 Accepted Papers Data Set”, Mendeley Data, v2 https://doi.org/10.17632/wj5vb6h9jy.2 Usage rights: Creative Commons Attribution 4.0 International license (CC BY 4.0)
Related research article	D. Vallejo-Huanga, P. Morillo, C. Ferri, Semi-Supervised Clustering Algorithms for Grouping Scientific Articles, Procedia Computer Science 108, pp. 325–334, (2017). https://doi.org/10.1016/j.procs.2017.05.206[2].

**Table:** 

**Value of the data** • The information of the dataset could be used for different applications such as building prototype systems for different tasks in the domain of Machine Learning or Information Retrieval such as: clustering analysis [2], multivariate querying, density estimation, testing and matching with similar datasets, categorization of papers using the topics of thematic sessions in which the papers were distributed and for other related tasks. • Another possible value of data could be to analyze the most popular topics in the field of Machine Learning.

## 1 Data

This data set is formed by metadata of the accepted papers in the *“International Conference on Machine Learning and Applications – ICMLA”* in the years 2014, 2015, 2016 and 2017. The dataset is in CSV format where each row is a paper (instance) and each column represents an attribute of that paper. The multivariate dataset contains 448 instances and 6 attributes. The dataset is part of the Mendeley Dataset Repository [1]. The attributes are: paper ID, title, keywords, abstract, session and year. The attribute format, description and size of each component of the dataset are summarized in Table 1.

**Table 1:** Description of dataset attributes.

	Format	Description	Minimum number of tokens	Maximum number of tokens
Paper_Id	Numeric	Identifier of the paper	1	1
Title	Free Text	Title of the paper	3	22
Keywords	Free Text	Author-generated keywords	1	11
Abstract	Free Text	Paper abstracts	54	315
Session	Categorical	Conference session in which the paper was exposed. Conference organizer's-selected.	1	11
Year	Numeric	Year of the conference	1	1

## 2 Experimental design, materials and methods

In the context of information retrieval [3], this dataset can be considered as a data collection of documents, where each instance is a document (scientific article) and attributes represent metadata.

A scientific article is usually composed of several sections that are distributed according to the type of journal or conference, author's style, thematic addressed, etc. Most scientific papers in machine learning area are prepared according to a format called IMRaD (Introduction, Materials and Methods, Results and Discussion). This format recommends a pattern or format rather than a complete list of headings or components of research papers. Other parts of a paper are: Title, Authors, Keywords, Abstract, Conclusions, and References. Additionally, some papers include Acknowledgments and Appendices [4].

The first attribute of the dataset is the identifier number of the paper, employed to distinguish the 448 scientific articles of the conference. Next, three attributes (title, keywords and abstract) were extracted by web scrapping from the IEEE Xplore Digital Library [5], through the Python Beautiful Soup library, and consolidated in a CSV file.

Beautiful Soup is a Python package for pulling data out of HTML and XML files. It works with multiple parsers to provide idiomatic ways of navigating, searching, and modifying the parse tree of the HTML/XML documents. We employed version 4.6.0 of Beautiful Soup, available for Python 2.6 + and Python 3+ [6].

PDFMiner is a Python-based solution to extract the text from a PDF. The tool is focused entirely on getting and analyzing text data and allows to obtain the exact location of given texts in a page, as well as other information such as fonts or lines. The solution also includes a PDF converter that can transform PDF files into other text formats (such as HTML). It has an extensible PDF parser that can be used for other purposes instead of text analysis [7].

The fifth attribute of this dataset has information about the organization of the thematic sessions of the conference in which the scientific articles were presented. The sessions were obtained by pdf scraping directly from the official page of the conference [8], through the Python PDFMiner library. The final distribution of the sessions includes the papers of the main conference, workshops and special sessions**.** This information was extracted in a new separated CSV file.

**Fig. 1:**
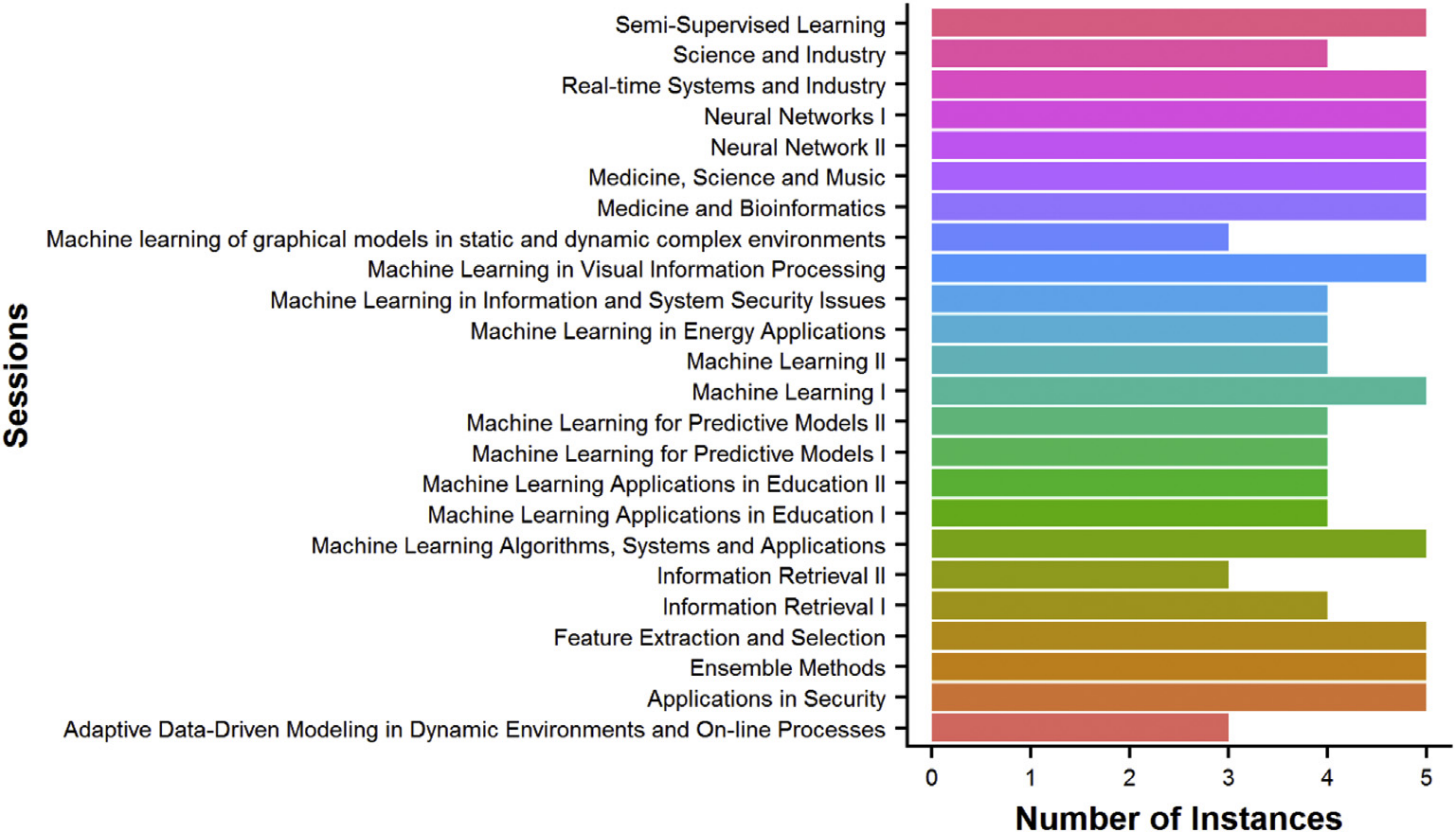
Configuration of thematic session**'**s vs. number of instances in ICMLA 2014.

To illustrate distribution of sessions with respect to the papers presented, a graph of the configuration of the sessions versus the number of instances in the ICMLA 2014 conference is shown in Fig. 1.

Finally, the last attribute of the dataset indicates the year of the conference. This attribute was placed manually in the dataset. Table 2 summarizes the amount of documents and sessions in the four editions of the conference compiled by this dataset.

**Table 2:** Distribution of documents and sessions in each year of the conference.

Year	Number of documents	Number of sessions
2014	105	24
2015	131	26
2016	108	24
2017	104	22

**Fig. 2:**
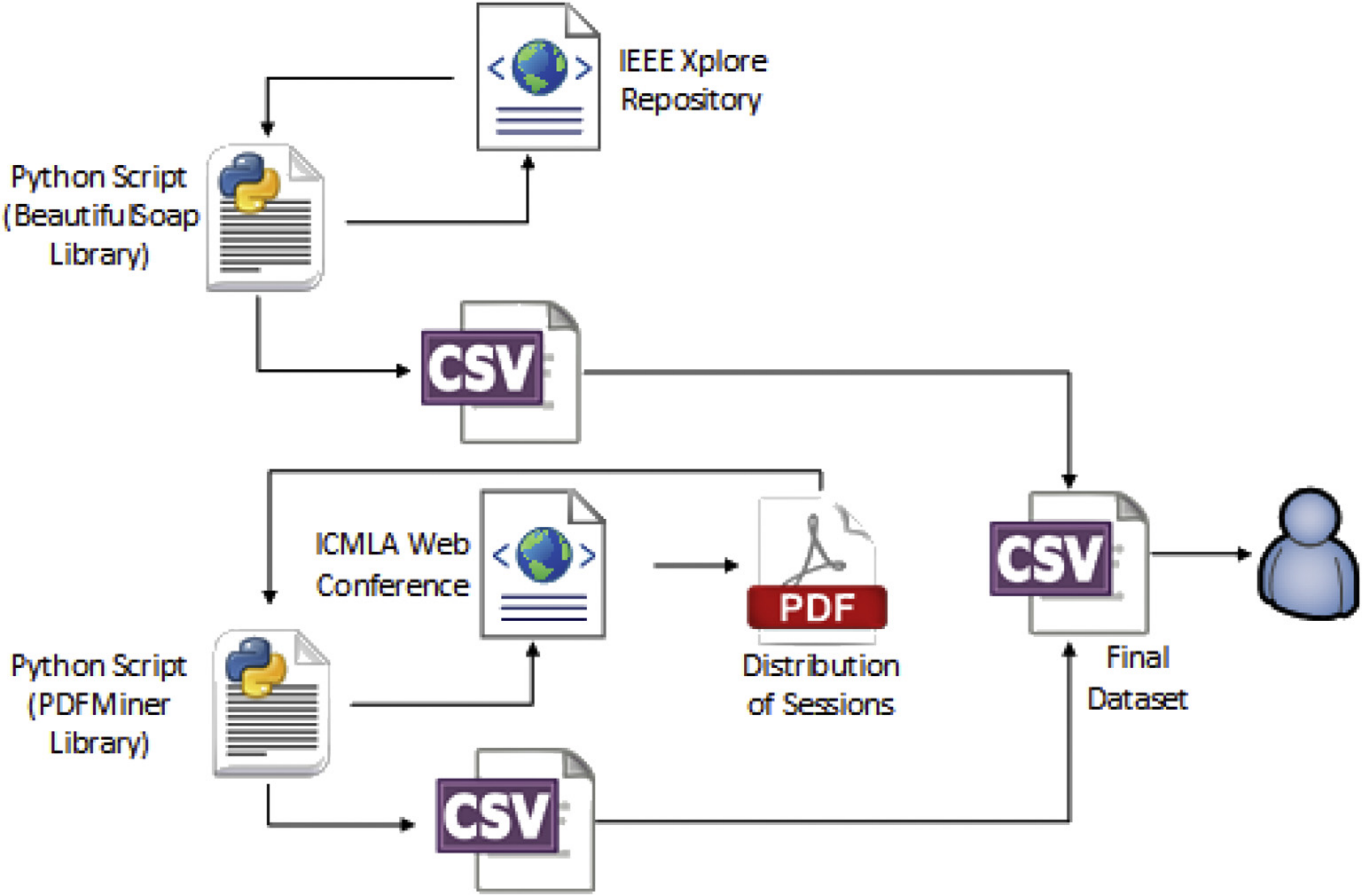
Data extraction scheme.

In this way, we obtained two CSV files: the first one with information about each paper, and the second one with the distribution of papers into sessions. The two CSV files were unified and homogenized for obtaining a single and final CSV file with the assembled dataset*.* Fig. 2 shows the data extraction scheme used for the construction of the dataset.

Natural Language Processing (NLP), can be defined as the study of mathematical and computational modeling of language [9], where the basic units, are the words [10]. Given the intrinsic complexity generated by any NLP process, many of the tasks are interdisciplinary related to other areas such as artificial intelligence, computer science, linguistics, etc. In NLP, a corpus is a collection of pieces of language text in electronic form, selected to represent a language or language variety [11].

Given a character sequence and a defined document unit, in NLP, tokenization is the task of chopping it up into pieces, called tokens, throwing away at the same time certain characters, such as punctuation [3], i.e., a token can be defined as a whitespace-separated unit of text and a document is an ordered collection of tokens [12]. In English, it is relatively easy to recognize the tokens since their delimiters are represented by space marks [10].

**Fig. 3:**
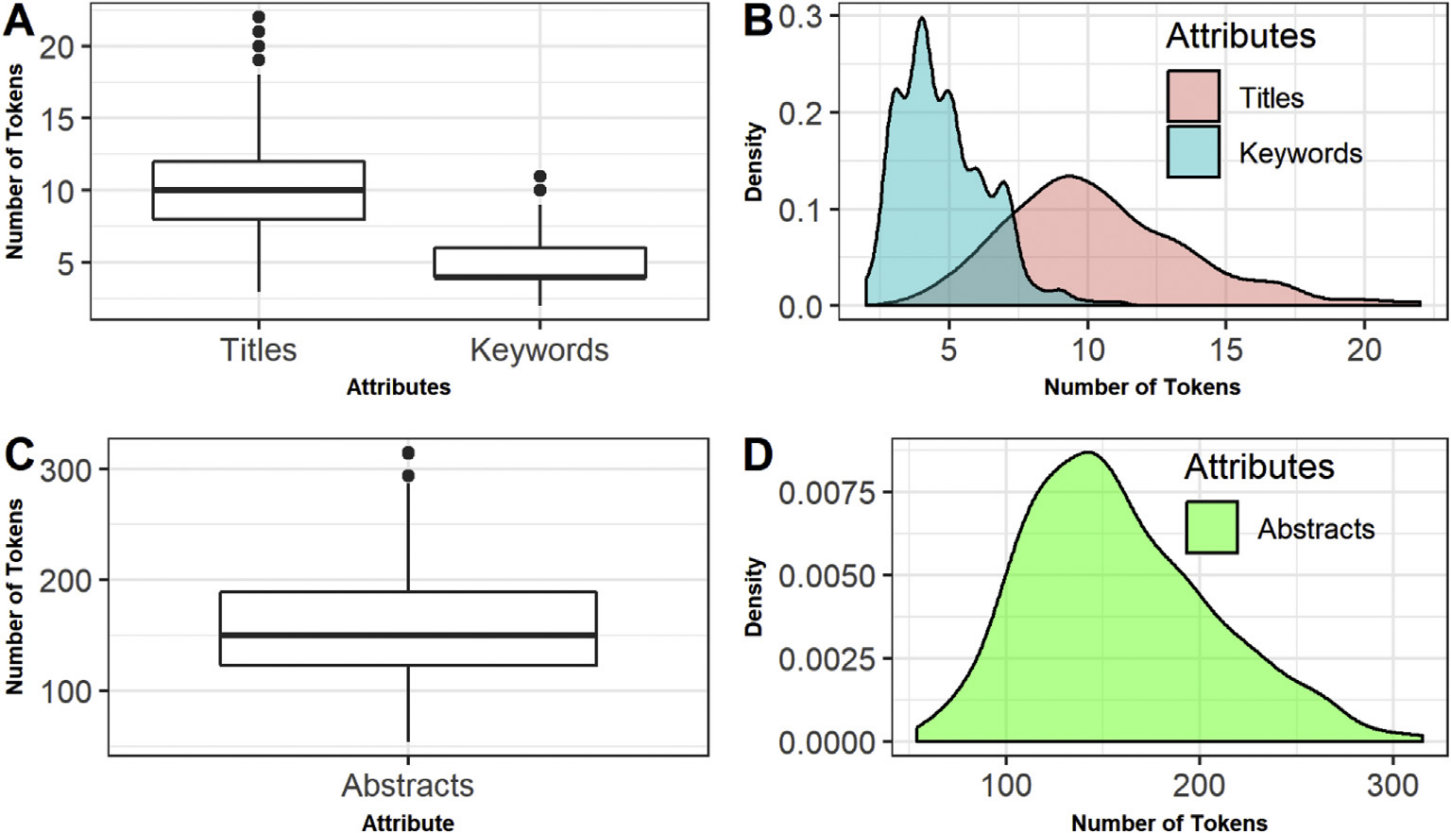
Distribution of the number of tokens.

Then, in this dataset each attribute of each instance is a text corpus in English language with a certain number of tokens, except the identifier number of the paper and the year of the conference. The session attribute can be considered as the class of the instance, if we want to consider the problem of clustering papers into thematic sessions. Some characteristics of the distribution of the tokens are shown in Fig. 3.

The top left of Fig. 3(A) shows the boxplot of the number of tokens of each paper with respect to the attributes: titles and keywords. The interquartile range (IQR) value for the title attribute and the keyword attribute is respectively 4 and 2. The graph at the top right Fig. 3(B) shows the density distribution of the number of tokens in the title attribute and in the keywords attribute. The bottom of Fig. 3 shows the boxplot (C) and the density distribution diagram (D) of the number of tokens of the abstract attribute. In this case, the IQR value is equal to 66.
